# Re: Clinical experience across the fetal‐fraction spectrum of a non‐invasive prenatal screening approach with low test‐failure rate

**DOI:** 10.1002/uog.22104

**Published:** 2020-09-01

**Authors:** R. Jelsema, Z. P. Demko, P. R. Billings

**Affiliations:** ^1^ Natera, Inc San Carlos CA USA

Hancock *et al*. report their clinical experience with a non‐invasive prenatal screening (NIPS) assay for fetal trisomy 21, trisomy 18 (T18) and trisomy 13 (T13) with a focus on cases with cell‐free DNA fetal fraction (FF) below 4.0%^1^. They present inferred sensitivity and inferred specificity metrics, and assert that their NIPS approach ‘performs comparably at high and low FF, rendering test failures due to low FF unnecessary’. We believe their conclusions are flawed for two main reasons.

First, accurate calculation of sensitivity and specificity requires the true outcome to be known for all tested samples, otherwise, ascertainment and reporting biases can distort estimates of performance^2^, ^3^. Hancock *et al*. report follow‐up for only 5.7% of their screen‐negative patients, however, they project the test performance of their assay based on the assumption that all false‐negatives (FN) have been reported and thus the FN response rate is 100%. This assumption is unfounded and not evidence‐based. FN results may not be reported by clinicians for many reasons, such as litigation concerns, patient confidentiality or prenatal care provider unawareness.

Second, Hancock *et al*. claim that their NIPS approach performs comparably at high and low FF, thus concluding that low FF does not affect the accuracy of their NIPS assay^1^. However, the inferred sensitivity shown in table S3 of their study^1^ (although we disagree with the assumptions in their calculation) indicates an observed FN rate of 1.1% for patients with FF ≥ 4% and 10.3% for those with FF < 4%, which contradicts directly their claims. This difference cannot be explained by a difference in follow‐up, as the response rates are quite similar between the two groups. Reanalysis of the data using an updated algorithm (table 3), as well as a theoretical demonstration of the impact of arbitrary changes in the response rates on test performance (table S1), still show differences in the performance of their NIPS approach for the FF ≥ 4% and FF < 4% groups^1^.

There are several other concerning inconsistencies in this study^1^. For example, the authors claim that their NIPS test has a negative predictive value of 100% for T13 despite the fact that the cohort contains two T13 FNs. Also, test performance is misrepresented in figure 2 of the study, as the authors represent both ‘likely’ and ‘confirmed’ screen negatives but only the ‘confirmed’ screen positives. Additionally, the performance of the updated algorithm presented in the study was based on a *post‐hoc* analysis of samples and was not confirmed in a prospective cohort.

Committee opinions from professional societies on cell‐free DNA screening for fetal aneuploidy state clearly that FF measurement is necessary for accurate test results, based on evidence from several studies^4^, ^5^. Low FF is associated with increased risk for T18, T13 and triploidy, and the use of a NIPS assay with suboptimal performance at low FF is highly problematic. Hancock *et al*. do not discuss triploidy as their technology is not designed to detect it.

In summary, the study findings of Hancock *et al*. do not support their claims regarding the performance of their NIPS test, especially in patients with lower FF. Alternative strategies are needed to ensure high accuracy for detecting common aneuploidies at low FF, such as test failure, redrawing at a later gestational age and adjusting risk scores based on FF.

### Disclosure

All authors are employees of and have stock options at Natera, Inc.
